# Exploiting Translation Machinery for Cancer Therapy: Translation Factors as Promising Targets

**DOI:** 10.3390/ijms251910835

**Published:** 2024-10-09

**Authors:** Urmila Sehrawat

**Affiliations:** Cancer Biology and Genetics, Memorial Sloan Kettering Cancer Center, New York, NY 10065, USA; sehrawu@mskcc.org

**Keywords:** translation initiation, drug target, eIF4F, eIF1, cancer therapeutics, small-molecule inhibitors

## Abstract

Eukaryotic protein translation has slowly gained the scientific community’s attention for its advanced and powerful therapeutic potential. However, recent technical developments in studying ribosomes and global translation have revolutionized our understanding of this complex multistep process. These developments have improved and deepened the current knowledge of mRNA translation, sparking excitement and new possibilities in this field. Translation factors are crucial for maintaining protein synthesis homeostasis. Since actively proliferating cancer cells depend on protein synthesis, dysregulated protein translation is central to tumorigenesis. Translation factors and their abnormal expressions directly affect multiple oncogenes and tumor suppressors. Recently, small molecules have been used to target translation factors, resulting in translation inhibition in a gene-specific manner, opening the door for developing translation inhibitors that can lead to novel chemotherapeutic drugs for treating multiple cancer types caused by dysregulated translation machinery. This review comprehensively summarizes the involvement of translation factors in tumor progression and oncogenesis. Also, it sheds light on the evolution of translation factors as novel drug targets for developing future therapeutic drugs for treating cancer.

## 1. Introduction

The expression of genetic messages is a crucial fundamental process and provides deep insights into the phenotypes of eukaryotic cells. Gene expression is extensively regulated at all stages, from transcription to protein synthesis during translation [1]. It is evident that mRNA translation is a significant stage of gene expression control [2] and maintains homeostasis for the expression of available cellular mRNA pools [3]. The translation process is generally divided into the following four steps: initiation, elongation, termination, and ribosomal recycling [1]. The primary translation control that regulates protein expression happens during the rate-limiting stage of the translation initiation step, wherein the small subunit of ribosome (40S) associates with translation initiation factors to recognize the AUG start codon on the mRNA template for a systematic sequential assembly of 80S elongation competent ribosome [2,4].

**Figure 1:**
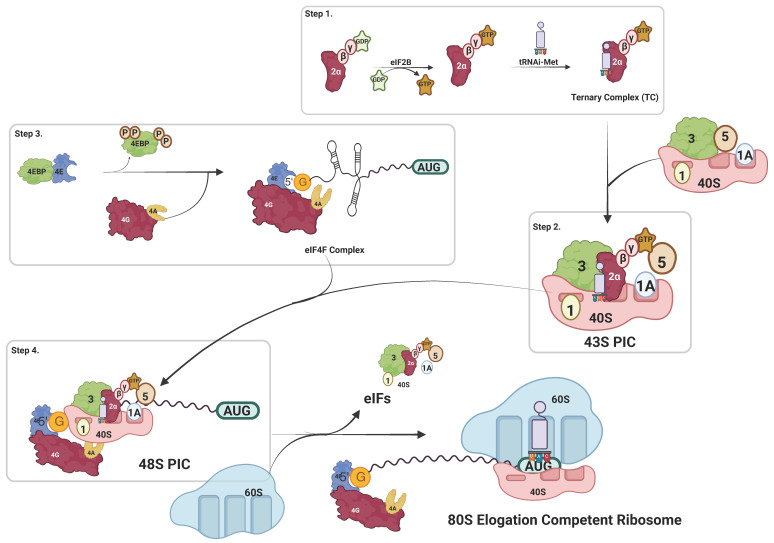
**Schematic representation of eukaryotic translation initiation**. Translation initiation is a multi-step process. In step 1, the ternary complex (TC) is formed wherein eIF2 (α, β, and γ), initiator methionyl tRNA (Met-tRNAMeti), and GTP assemble. In step 2, 40S ribosomal subunit, ternary complex, eIF1, eIF1A, eIF3, and eIF5 assembles to form 43S pre-initiation complex PIC). Simultaneously, the eIF4F complex consisting of eIF4E, eIF4A and eIF4G, binds to 5’cap-mRNA (step 3). Next, the 43S PIC is recruited onto the mRNA (step 4). eIF4A helps release mRNA secondary structure in 5’UTR and help facilitate pre-initiation 43S complex scanning of 5’UTR in 5’ to 3’ direction. Upon the AUG start codon recognition, all the translation factors (i.e., eIFs) are released that follows the joining of a 60S large subunit, leading to the formation of elongation-competent 80S ribosome marking the end of the translation initiation stage of translation.

Eukaryotic translation initiation is a multistep process that has been well characterized and believed to be the most prominent mechanism during the translation process. Briefly, during translation initiation, there is the simultaneous formation of 43S pre-initiation complex (PIC) (Figure 1, step 2), which is a multifactorial complex formed by the association of 40S ribosomal subunit with eukaryotic translation initiation factors (eIFs), i.e., eIF1, eIF1A, eIF3, eIF5, and ternary complex (TC). The ternary complex (TC) is a trimeric complex that includes eIF2 (containing subunits, i.e., α, β, and γ), initiator methionyl-tRNA, and GTP (Figure 1, step 1). The recruitment of 43S PIC to the mRNA is enabled by the eIF4F complex (Figure 1, step 3); the eIF4F complex consists of mRNA cap-binding factor eIF4E, a large scaffold factor eIF4G, and the DEAD-box helicase eIF4A. The eIF4F guides the recruitment of 43S PIC onto the mRNA through mRNA cap recognition via eIF4E and eIF4G-eIF3 interactions, forming the 48S initiation complex (Figure 1, step 4). The eIF4A helicase initially interacts with 5’UTR of mRNA and releases mRNA secondary structure, facilitating mRNA scanning by the 43S PIC towards the AUG start codon. The scanning process, which is critical to translation initiation, is stringently controlled by eIF1 and eIF1A; both these factors also facilitate AUG recognition by the 48S PIC; recognition of the start codon triggers the release of eIFs (i.e., initiation factors) and the joining of the 60S ribosomal subunit. In the final step, the formation of translation elongation competent 80S ribosome marks the end of the translation initiation stage and the beginning of the elongation stage (Figure 1).

The most critical step in translation initiation is the selection of the AUG start codon. The efficient translation initiation depends on the context of nucleotides surrounding AUG and the features of the 5’ untranslated region (5’UTR). The canonical context of nucleotides for translation initiation in mammals is KOZAK sequence (A/G)CCAUGG, where purine at position −3 and guanine at position +4 with reference to Adenine at position +1 is essential for stable interaction of mRNA and 48S complex [4,5,6,7]. Besides this, certain other features in 5’UTR of mRNA can influence translation initiation; for instance, 5’ UTR length should be at least 32 nucleotides long for efficient start codon recognition in a consensus context. Any deviations from the consensus context, shortening of 5’UTR length, and presence of additional AUG codons or near-cognate start codons in sub-optimal contexts in 5’UTR may give rise to inefficient translation initiation. For example, the presence of extra AUG in a suboptimal context in 5’UTR and 5’UTR shorter than ~20 nt may give rise to leaky scanning due to stearic clash between the cap complex and 43S complex [6,7], in which 43S PIC bypass the first AUG and translation initiates at the downstream start codon i.e., AUG, leading to formation of truncated proteins or different isoforms of polypeptides [8]. Notably, the secondary structures in the mRNA 5’UTR influence the translation initiation most. These distinct secondary structures include hairpins, long-range interactions, pseudoknots, R-loops, internal ribosomal entry sequences (IRES), and G-quadruplexes. Their presence impacts the initiation process negatively by slowing down the scanning process. The DEAD-box helicase eIF4A is an essential RNA helicase that unwinds these mRNA secondary structures, allowing scanning and thus facilitating translation initiation [9,10]. Most oncogenes, like MYC, RAS, BCL2, PIM1, etc., have secondary structures in their 5’UTR and, therefore, depend on eIF4A for their expression, suggesting a more stringent translation control for their oncogenic expression [11].

Translation dysregulation is considered a hallmark of cancer and is directly linked to abnormal survival, proliferation, angiogenesis, cancer metabolism, and altered immune response [2,12,13,14,15]. Translation control thus maintains the cellular proteome that ultimately regulates the gene expression. Most cancer-related mutations are linked to pathways that merge into translation machinery. Some of the most common oncogenes, like MYC, RAS, and PI3K, and tumor suppressors like TP53 and PTEN, are known to affect translation machinery components [16,17,18,19]. Therefore, it is essential to further deepen our understanding of molecular events of protein synthesis in cancer to develop effective cancer therapeutics. To this end, the mechanism of various translation factors in promoting malignancies and tumorigenesis has triggered wide attention in recent years to target the translation process therapeutically. The promising progress in targeting these factors to develop specific cancer therapeutics is a beacon of hope, providing a therapeutic window for selectively targeting cancer cells. This perspective review delves into the status of translation factors that cause multiple malignancies and the development of small molecules that bind directly to elements of the eukaryotic translational machinery. (see Table 1).

## 2. Translation Initiation Inhibition

The goal of discovering and developing translation inhibitors is to find mRNA transcript-level selectivity to block the expression of unwanted proteins while maintaining healthy cellular proteome homeostasis. This can be carried out by targeting the specific translational machinery components that differentially regulate protein expression in healthy and disease states or targeting specific mRNA−protein or protein-protein interactions during translation or in translation machinery. Therefore, identifying these targets in translational machinery, which is involved in regulating disease states, is crucial. Targeting these potential therapeutic targets using small molecules, RNA-silencing, or similar state-of-the-art approaches is essential for developing these translation factors as drug candidates with clinical applications.

Since initiation is the essential and highly regulated rate-limiting step, translation initiation factors are the main focus of developing the most targeted therapies targeting translation machinery. The urgency and importance of developing translation inhibitors can be underscored by the attempts to target the components of the eIF4F complex directly [46,67,68,69]. Other translation factors and their interactions have also been targeted since they are dysregulated in multiple malignancies, suggesting their prime role in cell transformation and tumor progression (Table 1). This section discusses individual translation initiation factors, their involvement in cancer onset, and the recent developments toward therapeutically targeting them to develop novel anticancer chemotherapeutic strategies.

### 2.1. eIF1 and eIF1A

eIF1 and eIF1A facilitate mRNA scanning during translation initiation [8,70]. Ribosomal biochemical and structural analysis has determined that the eIF1 and eIF1A bind 40S ribosomal subunits near P and A sites, respectively, and determine an open 40S conformation that is scanning competent [8]. eIF1 binding to ribosome leads to open conformation, and its dissociation from the pre-initiation complex triggers AUG recognition by the Met-tRNA interface [71,72], stabilizing the ribosomal closed conformation. In the case of eIF1A, the eIF1A C-terminal tail (CTT) enables the ribosomal open conformation. In contrast, closed scanning arrested conformation of the ribosomal 40S subunit is enabled by the N-terminal tail (NTT) of eIF1A. Therefore, the two tails of eIF1A play opposite roles during scanning and AUG recognition [73,74,75]. Additionally, eIF1A interacts with 43S PIC via ribosomal proteins RPS3 and RPS10 located at the ribosomal A site, which undergoes conformational changes during ribosomal subunit joining and 80S ribosome formation [76].

Recently, eIF1 has been implicated in hepatocellular carcinoma, wherein it was found to be overexpressed and linked to poor prognosis [20,21,22]. Targeting eIF1 directly is complicated due to its small size. However, eIF1 has recently been reported to interact with scaffold factor eIF4G1 [77]. The eIF1-eIF4G1 interactions are linked to scanning-dependent mRNA translation and maintaining ER stress response and mTOR-activated gene expression [63]. The eIF1-eIF4G1 complex has been targeted using small-molecule inhibitors, i.e., i14G1-10 and i14G1-12, which are effective in killing multiple cancer cell lines [78]. However, to successfully implement these compounds as anti-cancer therapeutics, further development of these eIF1-eIF4G1 complex inhibitors, i.e., i14G1s, is required.

eIF1A is a 17 KD initiation factor highly conserved among all eukaryotes. eIF1A tails help interact with other initiation factors during the translation initiation process. eIF1A interacts with eIF2, eIF3, and eIF5 through its N-terminal tail (NTT), while the C-terminal tail (CTT) helps interact with eIF5B and ternary complex (GTP-eIF2-initiator tRNA). Somatic mutations in eIF1A are associated with uveal and thyroid cancer and coexist with other mutations like Ras cancer [23,24,25,79]. The most frequent and recurrent hotspots, eIF1A mutations, occur on its exon 2 and intron splice sites. The mutations in exon 2 disrupt the N-terminal tail (NTT) of eIF1A, affecting its ability to promote the closed conformation of pre-initiation complex PIC and preventing leaky scanning [79,80]. On the other hand, 5’ splice site mutations alter the C-strand and C-terminal tail (CTT) domain of eIF1A [24]. Since eIF1A is a small protein, it is difficult to target it alone using small molecules. The best and most promising way to target eIF1A’s activity is perhaps by targeting its interactions with ribosomes and interlocking it in its scanning incompetent state.

Most oncogenic mRNA associated with cell proliferation and cell cycle depend on eIF1A and eIF1 for mRNA scanning and protein expression. Therefore, targeting these translation factors alone or in their interactive complexes provides a platform to block the expression of mRNAs highly dependent on mRNA scanning, suggesting potentially improved cancer therapies for cancer involving upregulation or dependencies on eIF1 and eIF1A.

### 2.2. eIF4A

eIF4A (eukaryotic initiation factor 4A) is the ATP-dependent DEAD helicase required to unwind the secondary structures in 5’UTR during 43S PIC mRNA scanning [11,81]. eIF4A’s helicase activity undergoes constant cycling between open and closed ribosome conformations. While ATP binding and mRNA induce closed conformation, ATP hydrolysis and mRNA disengagement lead to open conformation. The ratio of eIF4A to the ribosome is 3:1, making it one of the most abundant initiation factors necessary for cap-dependent translation initiation [15,82]. eIF4A has been shown to play a critical role in viral replication and tumor progression, suggesting broader applications of inhibiting eIF4A activity. Most oncogenic mRNA possesses long and highly structured 5’UTR that requires eIF4A for scanning and subsequent translation initiation. Thus, making these oncogenes dependent on eIF4A helicase activity. Some of the most eIF4A-dependent genes include MYC, cyclin D1, PIM1, and BCL2, which lead to tumor survival, excessive cell proliferation, and cell cycle abnormalities [11]. Similarly, eIF4A is required for viral mRNAs with highly structured IRES in their 5’UTR. For example, coronavirus hijacks host eIF4A to translate its highly structured mRNA [83,84]. Similarly, influenza viruses and other positive-strand RNA viruses depend on eIF4A helicase activity [85]. eIF4A expression is higher in proliferating cells [86]. More eIF4A expressions are upregulated in multiple malignancies, including breast, lung, ovarian, endometrial, and cervical cancers [39,40,42,87].

The primary class of eIF4A inhibitors includes rocaglate. Rocaglamide or RocA clamps eIF4A at polypurine sequences with mRNA utilizing ATP, which inhibits the 43S scanning PIC and thus inhibits translation initiation [88,89]. The natural rocaglate silvestrol works by dimerizing eIF4A and RNA, leading to limited loading of eIF4A onto the eIF4F cap complex that, in turn, inhibits global protein translation [90]. Additionally, silvestrol selectively inhibits the translation of mRNA containing G-quadruplex sequence in its 5’UTR, which includes oncogenes like MYC, MDM2, and RUNX1 [9]. Rocaglates, including silvestrol, are highly potent in in-vitro cytotoxicity, but their pharmacokinetics have significant challenges. For example, silvestrol has a short half-life in plasma due to efflux from cell membrane pumps, which limits the clinical development of silvestrol [91,92,93]. Interestingly, the potent cytotoxic activity of eIF4A inhibition led to the development of a cascade of synthetic RocA derivatives, i.e., CR-1-31-B, that binds to eIF4A at Glu195 [94]. Another synthetic rocaglate is zotatifin, or eFT226, which stabilizes eIF4A binding to mRNA at polypurine sequences [95]. It has recently been used for treating breast cancer and implicated in clinical trials in combination with estrogen receptor (ER) inhibitors [96]. Besides being effective in cytotoxicity activity, eIF4A inhibitors have shown antiviral activities. Silvestrol has shown effective against various viruses, including Ebola virus, MERS-CoV (Middle Eastern Respiratory Syndrome coronavirus), and human coronavirus 226E [97,98]. Additionally, many other small molecules are active against eIF4A. A natural marine compound, Peteamine A (PetA), inhibits eIF4A by binding to it irreversibly. PetA induces closed conformation of eIF4A, leading to the loss of its helicase activity [99,100]. Another translation inhibitor associated with eIF4A is hippuristanol, which interacts with the C-terminal domain of eIF4A, blocking its helicase and RNA-dependent ATPase activity [69,101]. Since eIF4A uses ATP hydrolysis to switch between open and closed conformation to release secondary structures in mRNA. This transition is inhibited by hippuristanol, limiting the helicase activity of eIF4A [102].

eIF4A is the most targeted translation factor by small-molecule inhibitors. Although most eIF4A inhibitors have shown potential gene-specific translation inhibition in multiple cancer models, the major setback is the associated cytotoxicity with eIF4A inhibitors, which makes it challenging to develop eIF4A inhibitors as potential drugs. Therefore, making these small molecules more efficient by increasing their on-target efficiency to kill, specifically, cancer cells and limit systemic toxicity is essential for their successful implication as novel and potential chemotherapeutics.

### 2.3. eIF4E

eIF4E (eukaryotic initiation factor 4E) is an essential factor that binds to mRNA 5’cap [81,103]. It has a concave surface that directs binding to the m7G cap specifically. Regulation of eIF4E is essential to maintain all cap-dependent translation control [67]. eIF4E phosphorylation at S209 by MAP-kinase signal integrating kinases like Mnk1 and Mnk2 enhances its affinity for m7G cap structures, making MnK1/2 inhibitors a target for eIF4E and thus for translation inhibition [68]. On the contrary, eIF4E dephosphorylation causes reduced affinity and thus slows down the global translation and sometimes triggers alternative cap-independent translation initiation [104]. Since eIF4E interacts with small translation repressor proteins, 4E-binding proteins (4E-BPs, i.e., 4E-BP1, 4E-BP2, and 4E-BP3) at the same site that it uses to bind eIF4G [105], therefore, 4E-BPs compete with eIF4G for its binding to eIF4E, thereby inhibiting eIF4F complex assembly. 4E-BPs are phosphorylated upon mTOR activation that dissociates 4EBPs from eIF4E. Upon release, eIF4E associates with eIF4G, thus forming eIF4F complex [67,106,107].

eIF4E is essential for the cap-dependent translation of all nuclear mRNAs, yet there is a subset of mRNAs that are highly dependent on eIF4E activity; these mRNAs are categorized as eIF4E-dependent mRNAs and are involved in cell proliferation, survival, and tumorigenesis. These eIF4E-dependent mRNAs include cyclins, VEGF (vascular endothelial growth factor), PRPS2 (phosphoribosyl-pyrophosphate synthetase 2), MYC, and ODC (ornithine decarboxylase) [108,109,110,111,112]. Hence, eIF4E is one of the most promising targets for translation inhibition. The role of eIF4E in neoplasia is very well studied and established. Not only does eIF4E overexpression exhibit oncogenesis in vitro, but multiple-fold eIF4E overexpression has been documented in various cancers, including breast, lung, leukemias, lymphoma, colon, and head and neck squamous cell carcinoma [28,43,44,45,46,47,48,49,113]. Since eIF4E availability for the eIF4F complex is regulated by 4E-BPs, expression of non-phosphorylated 4E-BP1 mutants has been shown to constitutively bind to eIF4E and suppress cell proliferation and neoplastic growth [114,115,116]. eIF4E has been indirectly targeted through mTOR and MNK inhibition by targeting 4EBPs and eIF4E phosphorylation [68,116,117,118,119,120,121]. However, this discussion focuses on the direct inhibitors of eIF4E developed to target its cap-binding activity using synthetic inhibitors. 

Ribavirin is one of the first synthetic nucleosides, initially developed as an antiviral drug, and was one of the first direct inhibitors of eIF4E. Ribavirin was discovered to mimic m7G-cap and compete for direct binding to eIF4E to block eIF4E-cap complex, leading to selective anti-proliferative activity in cancer, e.g., ribavirin selectively inhibits the translation of eIF4E-dependent oncogenes, i.e., BCL2, MYC, MCL1, etc., but it does not affect eIF4E-independent housekeeping genes in acute lymphoblastic leukemia (ALL) [122]. Next, the m7G-cap analogs were developed to inhibit protein synthesis, e.g., 7-BnGMP inhibited m7G-eIF4E cap-binding activity, and to improve its activity, 7Bn-GMP prodrug 4Ei-1 was synthesized as a chemotherapeutic drug [123,124]. One important eIF4E inhibitor is the small-molecule thiazole 4EGI-1, which disrupts interactions between eIF4E and eIF4G1 [125,126]. 4EGI-1 not only inhibited eIF4G1 binding but also stabilized 4E-BP binding, leading to enhanced inhibition of eIF4E activity, which is required for tumor suppression [127]. 4EGI-1 has promising anti-neoplastic properties without systemic toxicity in breast cancer and melanoma xenograft models. It has also been shown to diminish the expression of cyclin D1, BCL2, and MYC oncogenes in non-small-cell cancer cells and glioma cells [128]. Another small molecule that inhibits eIF4E binding to both eIF4G1 and 4E-BP is 4E1RCat and 4E2RCat. Further, 4E1RCat partially prohibits 43S recruitment and thus 80S formation [129,130]. And 4E1RCat was reported to sensitize the doxorubicin-resistant lymphoma mice models in combination therapy with doxorubicin [131]. These attempts to block eIF4E activity alone or in combination with eIF4G have shown promising results and hold the potential to be developed further to eradicate cancer, wherein cap-dependent translation of specific genes plays a critical role. However, ensuring that the eIF4E activity for housekeeping gene expression is not affected while targeting gene-specific translation of oncogenes dependent on eIF4E activity is essential. This might help maintain the targeted effect with the most negligible adverse effects.

### 2.4. eIF4G1

eIF4G1 is the scaffold protein of the eIF4F complex and facilitates the cap-binding complex and 43S complex assembly to initiate the translation. eIF4G1 associates with eIF4A to activate its helicase activity and direct it to the proximal 5’UTR region [132,133]. Additionally, eIF4G1 possesses RNA binding activity and interacts with eIF4E, which helps tightly bind the eIF4F complex to the 5’cap [3]. eIF4G1 is critical in enhancing the eIF4E affinity for cap structure through allosteric interactions [134]. eIF4G1 actively promotes 43S recruitment by interacting with other initiation factors [135,136]. eIF4G1 actively stimulates the translation of most mRNAs. eIF4G1 is overexpressed and associated with poor prognosis in breast, cervix, lung, and nasopharyngeal carcinomas [50,52]. eIF4G1 interactions have been targeted by translation inhibitor 4EGI-1 to reduce eIF4G1-eIF4E interaction in human melanoma and breast cancer cell lines, showing anti-neoplastic activity, which was also confirmed in mice xenograft models [137]. Recently, as discussed before, novel inhibitors of eIF4G1-eIF1 interactions i14G1-10 and i14G1-12 are reported to inhibit the growth of multiple cancer cell lines in vitro, suggesting enhanced interest in inhibiting translation by inhibiting eIF4G1 activity and interactions with other translation initiation factors [78]. Targeting eIF4G1 could strategically inhibit the expression of not only eIF4E-dependent mRNAs but also scanning-dependent mRNAs by inhibiting its interactions with eIF4E and eIF1.

### 2.5. eIF2 (α)

eIF2 is the primary factor in forming the ternary complex (TC) consisting of Met-tRNA, which combines with a small ribosomal subunit and forms a 43S pre-initiation complex (PIC). It is a multi-subunit multimeric guanosine triphosphatase comprising eIF2α, eIF2β, and eIF2γ. eIF2 exists in two states, the GTP-bound active and GDP-bound inactive forms, and the GTP/GDP cycle is regulated through reversible phosphorylation of eIF2α (Figure 1; step 1) [103]. eIF2α phosphorylation leads to the inactivation of eIF2, causing translation inhibition and an integrated cellular stress response [138]. Conversely, cellular stress responses like oxidation, heat shock, nutrition deprivation, and hypoxia could lead to phosphorylation of eIF2α [139]. Due to its central role in maintaining cellular stress response, eIF2α is essential for cell survival, proliferation, tumorigenesis, malignancy, and metastasis [139]. One of the ways eIF2α led to cell transformation is by elevating TC levels, leading to uncontrolled translation initiation when mutated [140]. eIF2 has been reported to play a critical role in multiple cancer types like non-small-cell lung carcinoma, melanocytic neoplasm, gastrointestinal, brain, thyroid, lymphoma, and sarcoma [22,26,27,28,29,30,31,32].

eIF2α phosphorylation can promote cell growth or harm cellular fate, depending on the stress response stimulus, duration, and intensity. In mice, eIF2α defected in phosphorylation, leading to cell transformation, whereas its phosphorylation induces apoptosis, suggesting eIF2α phosphorylation as a potential strategy to develop cancer therapeutics [140]. One approach to target and promote eIF2α phosphorylation is through HRI kinase inhibition by BTdCPU, which has shown promising results in both in vitro and in vivo cancer model systems [141,142]. In another strategy, using phosphatase inhibitors such as salubrinal was effective in inhibiting eIF2α dephosphorylation and has shown synthetic lethal phenotype in vitro [143,144]. In early attempts, eIF2 was targeted by TC inhibition using brominated derivatives of fluorescein, NSC119889, and NSC119893, which prevent the binding of Met-tRNAi to eIF2 in vitro [145]. However, the efficacy of direct TC inhibition in vivo has not yet been established.

The implication of eIF2 and TC in cancer makes them potential targets for translation inhibition. However, it is difficult to develop selective inhibitors since both eIF2 and TC are required to translate all nuclear mRNAs. Nevertheless, the careful implication of targeting eIF2α phosphorylation could strategically overcome the risk of global translation inhibition and help develop novel translation inhibitors that can be used for anticancer therapies for eIF2-associated cancer types.

### 2.6. eIF3 Complex

eIF3 is the largest and most complex translation complex, consisting of 13 subunits, i.e., eIF3a-m, that assemble to constitute the eIF3 complex. eIF3 plays a critical role in all stages of the translation process. eIF3 promotes 43S pre-initiation complex assembly, scanning, and start codon recognition [8,146,147]. Though it is a translation initiation factor, it is implicated in termination and stop codon readthrough [148,149]. eIF3 can interact with IRES of specific viral mRNAs and promote cap-independent translation initiation [150]. Also, eIF3 interacts with 26S proteasome and assists in protein quality control. Abrupt differential expression and amplification of different eIF3 subunits have been implicated in carcinogenesis by increasing cell proliferation, cell cycle progression, and activating signaling pathways involved in cell transformation. Since each eIF3 subunit possesses a differential role in protein synthesis, multiple eIF3 subunits may be used as prognostic markers in the cancers in which they play oncogenic or tumor suppressor roles [151]. Most eIF3 subunits are overexpressed in several cancers except eIF3e and eIF3f. eIF3a is amplified and a prognostic factor in the lung, especially NSCLC (non-small-cell lung cancer). eIF3a is correlated with breast, colon, gastric, and cervical malignancies [34]. eIF3a is associated with bladder and prostate cancer [35]. eIF3d was found to lead to tumor progression in gallbladder cancer [152]. Another subunit, eIF3h, is amplified with MYC in breast cancer [33]. Also, eIF3h has been found to enhance tumorigenicity, invasion, and proliferation in hepatocellular carcinoma via TGF-β and MAPK pathways, making it a prognostic marker for hepatocellular cancer patients [37]. eIF3i overexpressed in multiple colon cancer cell lines, showing its involvement in colon adenocarcinoma [153]. Given the importance of eIF3 subunits in tumorigenesis and neoplasia, attempts have been made to treat them. For example, small molecule mimosine, NCE22, and NCE30 showed cytotoxicity on tumor cells in vitro as the inhibitor of eIF3a, making these compounds candidates for eIF3a regulation with the potential to become anti-cancer agents [154,155]. Similarly, CM16 was reported to target eIF3h and has shown an antiproliferative effect in vitro [156]. eIF3 subunits have versatile functions during translation and protein quality control, making them attractive for developing anti-cancer therapeutics. Yet, they are essential protein subunits for cellular proteome homeostasis, which makes it hard to target them without systemic toxicity. Relevant work targeting eIF3 is underway to develop anti-tumor therapies for clinical studies in different cancers linked with abrupt eIF3 expression.

### 2.7. eIF6

eIF6 is an essential translation factor required for translation and ribosomal biogenesis. eIF6 is important for anti-association activity; by binding with 60S ribosomal subunit, it prevents premature assembly of 60S and 40S ribosomal subunits [157]. It is partly (~30%) expressed in the nucleus, is associated with pre-ribosomal particles, and is required for 60S ribosomal biogenesis [158]. Multiple studies have reported involvement of eIF6 in tumorigenesis and cancer progression [159,160,161]. Furthermore, eIF6 has been shown to play a vital role as a driver and prognostic marker in multiple cancers, including breast cancer [29], colorectal carcinoma [162], ovarian adenocarcinoma [58], non-small-cell lung cancer [163], lung adenocarcinoma [164], esophageal carcinoma [165], hepatocellular carcinoma, and glioblastoma [166]. Pharmacologic inhibition of eIF6 activity is associated with metabolic rewiring related to hepatocellular carcinoma [167]. eIF6 inhibition is implicated to be effective in RAS mutated and mTOR-sensitive pathways [168,169]. Recently, novel inhibitors of eIF6-60S interactions have been discovered, namely eIFsixty-1 (clofazimine), eIFsixty-4, and eIFsixty-6 [170,171]. These inhibitors have been shown to prohibit cell growth in a dose- and cell-specific manner and affect global translation. eIF6 inhibition using these novel compounds has shown promising results in hepatocellular carcinoma in vitro [171]. eIF6 can thus emerge as another translation factor that could be targeted to improve outcomes in disease states caused by its dysregulation.

## 3. Translation Elongation Inhibition

**Figure 2:**
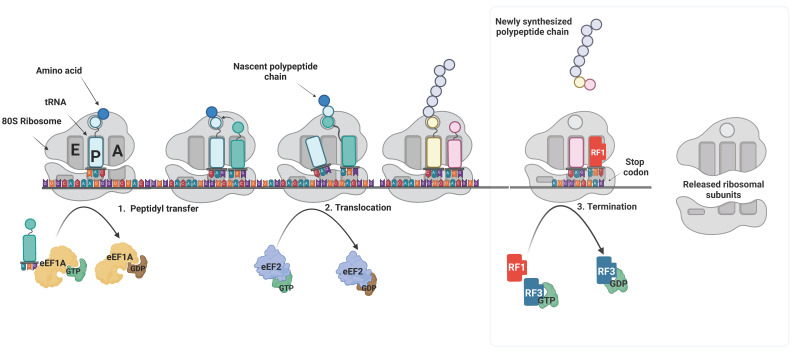
**Schematic representation of eukaryotic translation elongation followed by termination.** Translation elongation marks the peptidyl transfer reaction happening in the core of the ribosome. eEF1A brings amino-acyl-tRNA to the A-site of the 80S ribosome. eEF1A is released following GTP hydrolysis. In the next step, a peptidyl transfer reaction occurs by eEF2, wherein nucleophilic tRNA on the A site attacks the electrophilic peptidyl-tRNA in the P-site. Upon reaching the stop codon, eukaryotic release factors (RFs) bind to the A-site and allow the release of the completed polypeptide chain, marking the termination of the translation process.

Once the formation of 80S elongation-competent ribosome occurs, marking the completion of the translation initiation stage, the peptide synthesis continues when the eukaryotic elongation factor 1A (eEF1A) in its GTP-bound state binds and delivers aminoacyl-tRNAs (aa-tRNAs) to the A-site of the ribosome. The aa-tRNA delivered to the A-site forms a codon-anticodon interaction with the mRNA, followed by GTP hydrolysis, dissociating the eEF1A·GTP complex from the ribosome. In the next step, the A-site and P-site tRNAs are appropriately positioned to undergo peptidyl transfer in the peptidyl transferase center by catalytic bases in the 60S ribosomal subunit. In the following step, elongation factor eEF2, a translocase moves the ribosome along the mRNA to present the next codon so that polypeptide chain elongation can continue. The polypeptide chain is elongated until a stop codon is recognized. Eukaryotic release factors eRF1 and eRF3 are recruited at the stop codon site, which terminates the translation process and releases the new polypeptide chain (Figure 2). Multiple inhibitors that target elongation steps have been identified—most are directed to target eEF1A and ribosomes. Translation elongation inhibition development aims to enhance selective translation inhibition.

### 3.1. eEF1A

eEF1A is a crucial factor in the translation elongation stage of polypeptide synthesis. Higher vertebrates express two isoforms, eEF1A1 and eEF1A2, respectively. Human eEF1A1 is encoded by chromosome 6q14 and eEF1A2 by 20q13.3 [172]. These isoforms are differentially expressed across the tissues, while eEF1A1 is ubiquitously present in all tissues except the brain, heart, and skeletal muscles, where eEF1A2 is expressed predominantly [173,174]. Multiple malignant tissues express abnormal levels of both eEF1A1 and eEF1A2. Although eEF1A is centric to the elongation step of the translation process, leading to the expression of high-fidelity protein synthesis, it has also been found to be involved in multiple cellular processes, making it a unique translation factor. The cellular process that plays critical roles in signal transduction [175,176,177,178], apoptosis [179,180], cell migration [181], cytoskeletal organization [182], metabolism [183,184,185], neurodevelopment [173,174,186,187,188], post-transcriptional regulation [189], heat-shock response [190], and protein turnover [191,192].

The link between eEF1A and cancer was determined decades ago, with the findings that eEF1A indulgence triggers a transformation [193,194]. Later, eEF1A overexpression was noted in metastasis [195]. Initially, overexpression of eEF1A2 was correlated with anchorage-independent growth and in vivo tumorigenesis in ovarian cancer cell lines, and tumor samples were reported [59,60,64]. Later, many studies reported eEF1A in different cancer types in human cancer cell lines and tumor samples, including breast, lung, liver, prostate, pancreas, thyroid, hematological, kidney, and more (Table 1). Both eEF1A1 and eEF1A2 were reported as prognostic markers for multiple cancers such as renal cell carcinoma [196], pediatric AML [197], and colon adenocarcinoma [198,199]. A recent study used a CRISPR activation screen and reported eEF1A1 as one of the potential biomarkers to predict radiotherapy resistance in lymphoma [200]. All these evident studies highlight eEF1A as an oncogene primarily involved in the elongation step of the mRNA translation process. The oncogenic nature of eEF1A is attributed to its ability to drive malignancy through multiple oncogenic pathways, including PI3K/AKT pathway, mTOR, NF-KB, JAK/STAT, TGF-B, HIF1A, etc. Additionally, eEF1A also has been reported to inhibit tumor suppressor proteins and miRNA. eEF1A negatively impacted chemotherapy-induced apoptosis and p53, leading to chemoresistance to essential anti-cancerous drugs and suggesting strong involvement of eEF1A during anticancer drug resistance.

There have been attempts to target eEF1A using small molecules, i.e., didemnin B and plitidepsin (dehydrodidemin) are cyclic depsipeptide isolated from marine tunicate. The invitro activity of plitidepsin is in the lower nM range in most hematologic cancer cell lines (i.e., leukemia, lymphoma) and solid tumors (i.e., pancreas, breast, lung, and colon). Plitidepsin, in combination with dexamethasone, was approved for treating multiple myeloma in Australia for patients who have failed frontline treatments [201,202,203]. Several potential eEF1A inhibitory compounds have shown potent antiproliferative activity and efficacy in both in vitro and in vivo studies. These compounds include narciclasine, nannocystin, LQ18, and ansatrienin A [204,205,206,207]. Given the role of eEF1A in multiple vital cellular processes and oncogenic mechanisms, targeting eEF1A can significantly impact the development of cancer therapeutics, either alone or in combination therapies, to combat cancer and improve treatment strategies using novel chemotherapeutic approaches.

### 3.2. eEF2

eEF2 is the second important factor involved in the elongation of the polypeptide chain during translation. eEF2 is the center for translation control at the elongation step under multiple cellular stress conditions. It is overexpressed in gastric and colorectal cancer and related to cell cycle progression by activating AKT and G2/M-associated pathways [66,208]. In addition, eEF2 has also been found to overexpress in multiple other cancers, including lung, breast, prostate, glioblastoma, and hepatocellular cancer [66,209,210,211]. Interestingly, eEF2 was identified as a tumor-associated antigen, and eEF2-derived polypeptides are immunogenic and induce activated CD8+ T cells or cytotoxic T lymphocytes in vitro, suggesting potential adoptive transfer cancer immunotherapeutic for colon cancer [212]. This suggests that a promising role for eEF2 is being developed as a drug target to treat cancer, where it plays a crucial role.

## 4. Indirect Inhibitors of Translation

Although this review mainly discusses direct inhibitors of translation factors, several indirect translation inhibitors affect the activity of translation factors and ribosomal machinery during translation. Most of these translation inhibitors can be characterized as upstream signaling inhibitors. These include mTOR and MNK signaling pathway inhibitors, which affect eIF4F complex formation [118], and active inhibitors of mTORC1, including rapamycin, rapalogs, torin1, MLN0128, etc. Since mTOR phosphorylates 4E-BPs and allows eIF4F complex formation by releasing eIF4E from 4E-BPs sequestering. These mTOR inhibitors affect the availability of eIF4E and thus block eIF4F complex formation, leading to the direct effect of global translation [115]. On the other hand, MNK inhibitors (i.e., cercosporamide, CGP57380, retinamides, resorcylic acid lactones) inhibit eIF4E phosphorylation and have antiproliferative activity in multiple cancer cell lines [68,119]. Also, MNK inhibitors can help overcome chemotherapeutic resistance to rapamycin, herceptin, and gemcitabine [213,214,215,216]. Some other translation inhibitors that block ribosomal translation and have shown clinical evidence include homoharrigtonine and its analogs [217]. Homoharrigtonine prevents the formation of the first peptide bond and blocks the translation at the initiating ribosome. It has been approved for chronic myeloid leukemia [218]. It should be noted that translation factors can be targeted in combination therapies, where using indirect targets, such as MNK, mTOR, or ribosomes, could lead to synergistic effects and better outcomes in combating tumor progression.

## 5. Perspective

**Figure 3:**
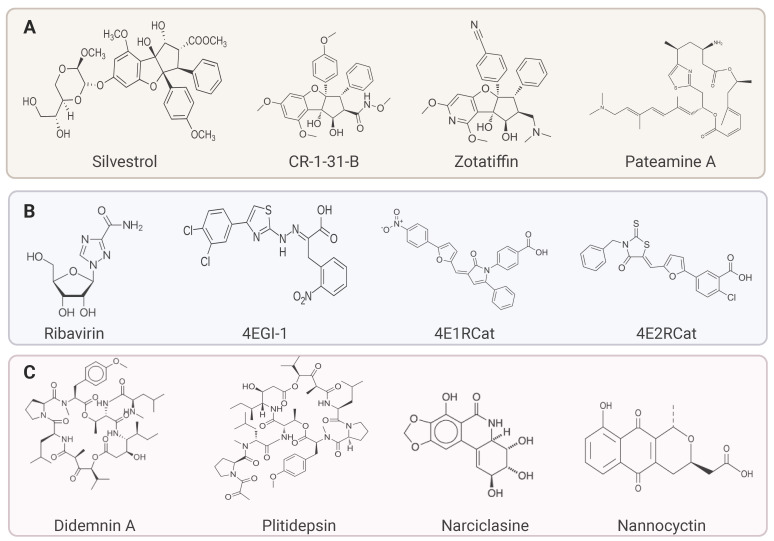
**Collection of selected translation inhibitors targeting different translation factors.** (**A**) eIF4A inhibitors: silvestrol and other synthetic Rocaglamide analog CR-1-31-B, Zotatiffin, and marine compound eIF4A inhibitor Pateamine A. (**B**) eIF4E inhibitors: ribavirin, 4EG1-I, 4E1Rcat, and 4E2RCat. (**C**) eEF1A inhibitors: didemnin A, plitidepsin, narciclasine, and nannocyctin.

**Figure 4:**
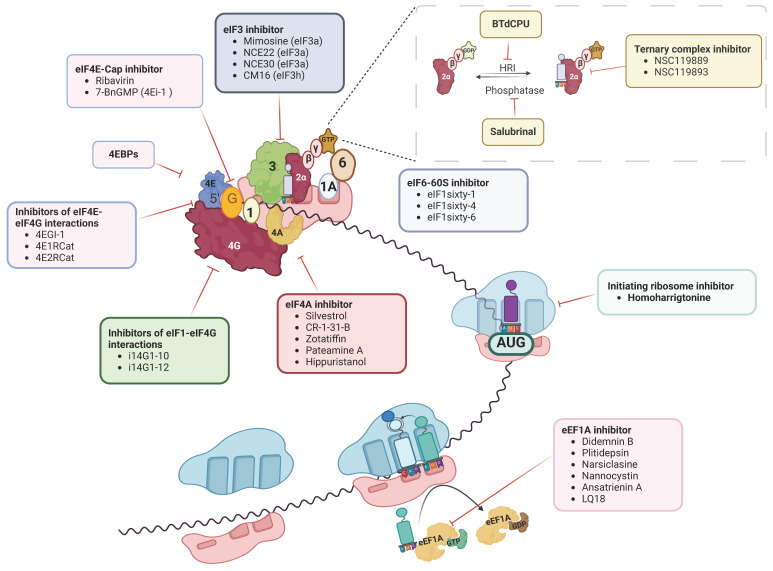
**Potential direct inhibitors of translation factors are being explored for developing novel cancer therapeutics.** Essential and potential drug target translation factors in the translation machinery and the compounds that target them are shown. Red bar-headed lines indicate inhibition. As shown, almost every translation factor in translation initiation can be targeted by small-molecule inhibitors, including eukaryotic translation initiation factor 2α (eIF2α) phosphorylation and ternary complex formation (upper left), eIF4E and eIF4F complex formation, enzymatic activity and cap-binding (upper right), eIF4A (center), eIF4G1 (center right), eIF3 (upper middle) as well as first peptide bond formation or initiating ribosome (center left) and translation elongation by eEF1A (bottom left).

mRNA translation is a substantial process that has been shown to play a critical role in cancer development. Recently, the development in understanding the role of translation factors in malignancies has provided essential evidence for developing translation inhibitors as promising therapeutics. Since most translation factors show aberrant expression and associated dysregulated translation, they are exceptional novel targets for cancer therapeutic development. Encouragingly, most translation factors in translation initiation and elongation can be targeted using small-molecule inhibitors (Figure 3 and Figure 4). Notably, most translation inhibitors include RNA in binding with their targeting factors, suggesting an essential role of RNA intercalators to be developed and tested towards inhibiting translation; for example, silvestrol targets eIF4A bound to mRNA polypurine sequence. Recent advancement of novel strategies to study translation, including ribosome profiling, cryo-electron microscopy, and single-molecule FRET (fluorescence resonance energy transfer), has played a vital role in the understanding and discovery of translation mechanisms using small-molecule inhibitors, which has eventually opened the door for these small molecules to be developed as potential drugs in cancer therapeutics, as it appears that the use of such small-molecule inhibitors to treat cancer is imminent and holds promise to minimize the problem of acquired resistance to available cancer chemotherapies.

However, transitioning translation inhibitors to the clinic will present new challenges unique to direct translation inhibitors due to associated systemic cytotoxicity. Because most translation factors are essential genes required for normal cellular homeostasis, the imbalance in their expression and activity leads to disease states like cancer development. However, targeting these factors could lead to overall cellular toxicity. With an enhanced understanding of the translation mechanism of each translation factor, it has become easier to develop novel strategies to target them or their interactions, both of which have shown promise in different in vitro and in vivo cancer models. Substantially, small-molecule translation inhibitors targeting different translation factors have demonstrated promising outcomes in preclinical and clinical stages (Table 2.). Interestingly, some translation inhibitors have gained clinical successes, such as Aplidin (Plitidepsin) in combination therapy with dexamethasone in multiple myeloma, and Zotatiffin (eFT226) has shown impressive results in solid tumors clinical trials. These advancements present a new avenue to develop and accelerate small molecules targeting translation. It is also evident that translation factors can be used in novel combination chemotherapeutic approaches by targeting them along with other cellular processes like immune checkpoints, signaling factors, cell cycle, etc. As discussed previously, a significant challenge to overcome in targeting translation machinery is the associated systemic toxicity, off-target activity, and inappropriate plasma half-life, which limits the development of these drug-like molecules in preclinical murine model systems (e.g., CR-31-B) and clinically in patients (e.g., Plitidepsin). These hurdles make it hard for these drugs to be advanced to the clinics. Therefore, it is crucial to determine additional ways to deliver these compounds to the local tumor site in optimal amounts, sufficient for killing cancer cells and eliminating systemic toxicity. The exhaustion of available drug targets in cancer and other disease states underscores the importance of leveraging new cellular mechanisms and pharmacologic modalities such as translation factors. Thus, targeting them could open new avenues for research and development in cancer therapeutics. The available data targeting translation factors suggest a promising strategy to tackle the therapeutic challenges in developing cancer therapeutics and tackle the ongoing quest for drug resistance towards available chemotherapeutic drugs. The upcoming successes of therapeutics that target dysregulated translation in cancer predict their transition from the bench to the bedside well.

## Figures and Tables

**Table 1:** A comprehensive overview of translation factors, their functions, and their aberrant expression in human cancer. This summary provides an understanding of the key players in translation and their potential as targets for cancer therapeutics.

Translation Factor	Function	Expression Discrepancy	Type of Cancer
eIF1	Maintains the integrity and stringency of start codon selection and mRNA scanning	Low expression	Hepatocellular Carcinoma, Pancreas Cancer [20,21,22]
eIF1A	Maintains mRNA scanning in cooperation with eIF1, helps start codon recognition, and prevents premature 40S joining.	Mutations	Uveal Melanomas, Thyroid, Ovarian, and Leptomeningeal Melanocytic Neoplasms [23,24,25]
eIF2A/B	Ternary complex (TC) formation with initiator tRNA, pre-initiation complex maintenance, and preventing 40S ribosomal subunit premature joining.	Overexpression	Non-small-cell Lung Carcinoma, Melanocytic Neoplasm, Gastrointestinal, Brain, Thyroid, Lymphoma, Sarcoma [26,27,28,29,30,31,32]
eIF3 complex	Organization and maintenance of the stability of 43S pre-initiation complex, prevention of premature binding between small (40S) and large (60S)ribosomal subunits.	Overexpression	Breast, Bladder, Cervix, Esophagus, Lung, Stomach, Colon, Ovary, Pancreas, Vulva, Prostrate, Hepatocellular Carcinoma [22,33,34,35,36,37,38]
eIF4A	It plays a crucial part in the eIF4F complex, is a critical player in translation initiation, and has an RNA helicase activity that unwinds secondary structures in mRNA’s 5’UTR.	Overexpression	Hepatocellular Carcinoma, Melanoma, Lymphoma, Breast, Lung [11,39,40,41,42]
eIF4E	Part of the eIF4F complex binds to mRNA 5’Cap. It stimulates eIF4A unwinding activity and recruitment of the pre-initiation complex.	Overexpression and hyper-phosphorylation	Breast, Bladder, Brain, Cervix, Colon, Liver, Lung, Head & Neck, Prostrate, Skin [28,43,44,45,46,47,48,49]
eIF4G1	It is the scaffold factor of the eIF4F complex, crucial for its assembly. It stimulates the helicase activity of eIF4A and the cap-binding efficiency of eIF4E. Its interactions with eIF1 facilitate mRNA scanning.	Overexpression	Breast, Cervix, Lung, Nasopharyngeal [50,51,52,53]
eIF5	This guanine transferase is required to activate multiple translation factors. It maintains a stable connection of ribosomal subunits during subunit joining or 80S ribosome formation.	Overexpression	Hepatocellular carcinoma, Lung, Glioblastoma, Cervix, Colorectal [54,55,56,57]
eIF6	Regulation of ribosomal biogenesis and premature binding of ribosomal subunits.	Overexpression	Colorectal, Ovarian, Leukemia, Head & Neck, Colon, Rectum, Pancreas, Lung [58]
eEF1A	Delivers all aminoacyl-tRNA to the ribosome and catalyzes the peptidyl transferase step during the elongation.	Overexpression	Blood, Breast, Lung, Liver, Prostrate, Pancreas, Thyroid, Kidney [51,59,60,61,62,63,64,65]
eEF2	Catalysis of ribosomal translocation step during elongation.	Overexpression	Gastric, Colorectal Carcinoma, Breast, Prostrate, Lung, Hepatocellular Cancer [66]

**Table 2:** Therapeutic direct inhibitors of translation in cancer. The table below lists translation inhibitors specific to translation factors and their development stage.

Translation Factor	Mechanism	Inhibitor	Development Stage
eIF1	eIF1 interaction with eIF4G1, blocking scanning and AUG recognition	i14G1-10 i14G1-12	Preclinical: activity in in vitro multiple cancer cell lines
eIF2α	eIF2α phosphorylation activation by HRI	BTdCPU	Preclinical: activity in xenograft models
eIF2α	eIF2α dephosphorylation inhibition	Salubrinal	Clinical: synthetic lethal in combination with proteasome inhibitor; Phase II clinical trials in combination with Carfilzomib (NCT01775553)
Ternary Complex	Met-tRNAi-eIF2 interaction inhibition	NSC119889, NSC119893	Preclinical: in vitro translation inhibition
eIF3a	Reduction in eIF3a expression	Mimosine, NCE22, and NCE30	Preclinical: in vitro cytotoxic activity
eIF3h	Inhibition of eIF3h activity	CM16	Preclinical: in vitro cytotoxic activity
eIF4A	Inhibition of eIF4A helicase activity	Silvestrol CR-1-31-B Zotatiffin Pateamine A Hippuristanol	Preclinical: excellent efficacy in multiple cancer xenograft models for silvestrol, CR-1-31-B, Pateamine A, and Hippuristanol Clinical: Zotatiffin (eFT226) is being studied in solid tumor malignancies (NCT04092673)
eIF4E	Inhibition of eIF4E-Cap interactions	Ribavirin 7-BnGMP (4Ei-1)	Preclinical: antiproliferative activity in vitro
eIF4E	Inhibition of eIF4E-eIF4G1 interactions	4EGI-1 4E1RCat 4E2RCat	Preclinical: antiproliferative activity in vitro and in vivo xenograft models
eIF4G1	Interaction with eIF1 and eIF4E blocking scanning and AUG recognition	i14G1-10 i14G1-12 4EGI-1	Preclinical: antiproliferative activity in vitro
eIF6	Interaction with eIF1 and 60S	eIF1sixty-1 eIF1sixty-4 eIF1sixty-6	Preclinical: antiproliferative activity in vitro and in vivo cancer models
eEF1A	Irreversible binding to eEF1A, blocking its peptidyl transferase activity	Didemnin B Plitidepsin Narsiclasine Nannocystin Ansatrienin A LQ18	Preclinical: antiproliferative activity in vitro and in vivo cancer models Clinical: Plitidepsin has been approved for treatment in multiple myeloma in combination with dexamethasone

## Data Availability

This study did not create or analyze new data, and data sharing does not apply to this article.
